# Mexiletine prevents transient heart failure in a polymicrogyria child with an ATP1A3 variant: a case report

**DOI:** 10.1093/ehjcr/ytag106

**Published:** 2026-02-06

**Authors:** Mai Aida, Junichi Ozawa, Yuya Takahashi, Satoko Miyatake, Kenichi Watanabe

**Affiliations:** Department of Pediatrics, Nagaoka Red Cross Hospital, 2-297-1, Senshu, Nagaoka, Niigata 940-2085, Japan; Department of Pediatrics, Nagaoka Red Cross Hospital, 2-297-1, Senshu, Nagaoka, Niigata 940-2085, Japan; Department of Pediatrics, Nagaoka Red Cross Hospital, 2-297-1, Senshu, Nagaoka, Niigata 940-2085, Japan; Department of Human Genetics, Yokohama City University Graduate School of Medicine, 3-9, Fukuura, Kanazawa-ku, Yokohama, Kanagawa 236-0004, Japan; Department of Clinical Genetics, Yokohama City University Hospital, 3-9, Fukuura, Kanazawa-ku, Yokohama, Kanagawa 236-0004, Japan; Department of Neurogenetics, Molecular Neuroscience Research Center, Shiga University of Medical Science, Seta Tsukinowa-cho, Otsu, Shiga 520-2192, Japan; Department of Pediatrics, Nagaoka Red Cross Hospital, 2-297-1, Senshu, Nagaoka, Niigata 940-2085, Japan

**Keywords:** *ATP1A3*, Na^+^/K^+^ ATPase, Polymicrogyria, Heart failure, Mexiletine, Case report

## Abstract

**Background:**

Several variants in *ATP1A3*, a gene encoding the α3-subunit of Na^+^/K^+^ adenosine triphosphatase (Na–K–ATPase), are reportedly involved in polymicrogyria. Some cases exhibit transient heart failure (HF); however, its underlying mechanism and prevention strategy remain unknown.

**Case summary:**

The patient was a 5-year-old female who suffered from intractable seizures since birth and was diagnosed with polymicrogyria based on a head magnetic resonance imaging scan. Whole-exome sequencing identified a *de novo* heterozygous *ATP1A3* variant, c.2976_2978del, p.(Asp992del). At 4 years of age, she experienced transient HF for the first time. Because bradycardia could trigger transient HF, we administered cilostazol. However, her bradycardia and subsequent transient HF recurred every few months. Therefore, mexiletine was initiated as an upstream therapy. During 1.5 years of follow-up, she has not experienced HF except once when she could not take mexiletine because of vomiting.

**Discussion:**

Loss of function of the Na–K–ATPase caused high concentrations of intracellular Na^+^ and subsequent increments in Ca^2+^ via the Na^+^/Ca^2+^ exchanger in the steady state. An augmentation of the late Na^+^ current (I_Na, L_) due to bradycardia may have led to further increments of intracellular Na^+^ and Ca^2+^, causing myocardial stunning. Based on the proposed pathophysiological mechanism, we selected mexiletine, which blocks Na^+^ channels particularly in the inactivated state, reducing I_Na, L_ and the subsequent Ca^2+^ overload. Mexiletine was administered safely, successfully preventing transient HF.

Learning points

- Several variants in *ATP1A3*, a gene encoding the α3-subunit of Na^+^/K^+^ adenosine triphosphatase, would cause polymicrogyria with transient heart failure (HF).
- Heart failure in *ATP1A3*-related polymicrogyria can be caused by myocardial stunning because of intracellular Ca^2+^ overload triggered by bradycardia complicated with seizures.
- Mexiletine may prevent transient HF in *ATP1A3*-related polymicrogyria by inhibiting the increase in Na^+^ current and subsequent Ca^2+^ overload via Na^+^/Ca^2+^ exchanger.

## Introduction

Na^+^/K^+^ adenosine triphosphatases (Na^+^/K^+^ ATPases) are transmembrane ion pumps that extrude three Na^+^ and import two K^+^ for every ATP split, regulating electrochemical gradients.^1^  *ATP1A3* encodes the α3-subunit of Na^+^/K^+^ ATPases expressed in neurons and cardiomyocytes.^1,2^  *ATP1A3* variants cause characteristic functional brain disorders; alternating hemiplegia of childhood (AHC) and rapid onset dystonia-parkinsonism (RDP) are caused by loss-of-function (LOF) variants.^3^ Reportedly, the most severe functional defects by *ATP1A3* variants which leads to the dysfunction of Na^+^/K^+^ ATPases were involved in polymicrogyria, which is extremely rare and has only been reported by a few patients.^4,5^ Some cases exhibited transient heart failure (HF); however, only cilostazol has displayed partial efficacy for HF prevention to date.^6^ Therefore, a mechanism of the transient HF and its prevention strategy should be elucidated. Herein, we report a case of a female child with polymicrogyria due to *ATP1A3* variants who suffered from recurrent transient HF, which was successfully suppressed by mexiletine.

## Summary figure

**Table:** 

Age	Event
Day of birth	She suffered from frequent generalized tonic–clonic seizures
4 months old	Whole-exome sequencing identified a *de novo* heterozygous *ATP1A3* variant, and she was diagnosed with polymicrogyria
4 years and 2 months	The first event of transient HF
4 years and 4 months	The second event of HF triggered by bradycardia; to prevent bradycardia, cilostazol was administered
4 years and 8 months	The third event of transient HF
4 years and 9 months	The fourth event of transient HF; mexiletine was initiated
5 years (the current)	The patient’s transient HF has been well-prevented

## Case presentation

**Figure 1:**
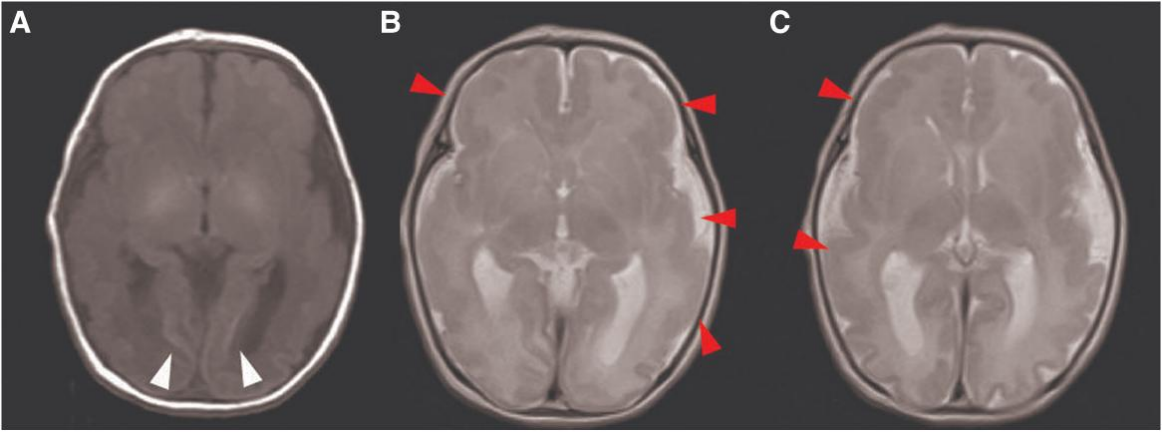
Brain magnetic resonance imaging scan performed on the day of birth. (*A*) T1-weighted images show high signal intensity at the corticomedullary border, indicating calcification (white arrow). (*B* and *C*) T2-weighted axial images depict polymicrogyria with cortical thickening extending from the parietal to temporal areas, predominantly in the frontal areas on both sides (red arrow).

The patient was a 5-year-old female who was born after 35 weeks of gestation through an emergency caesarean section because of fetal distress. Considering she was not breathing spontaneously at birth, she was managed on a ventilator. Additionally, she suffered from frequent generalized tonic–clonic seizures. Head computed tomography and magnetic resonance imaging scans identified polymicrogyria and calcification in the corticomedullary border zone (*Figure 1*). Whole-exome sequencing identified a *de novo* heterozygous *ATP1A3* (NM_152296.5) variant, c.2976_2978del, p.(Asp992del). The variant was confirmed as pathogenic based on the American College of Medical Genetics and Genomics guidelines for interpreting sequence variants (PS2 + PS3 + PM2).^7^ Considerably, she was diagnosed with polymicrogyria caused by the novel *ATP1A3* variant. Her seizures were intractable and required the combination therapy of multiple antiepileptic drugs. She was completely bedridden and could not communicate.

**Figure 2:**
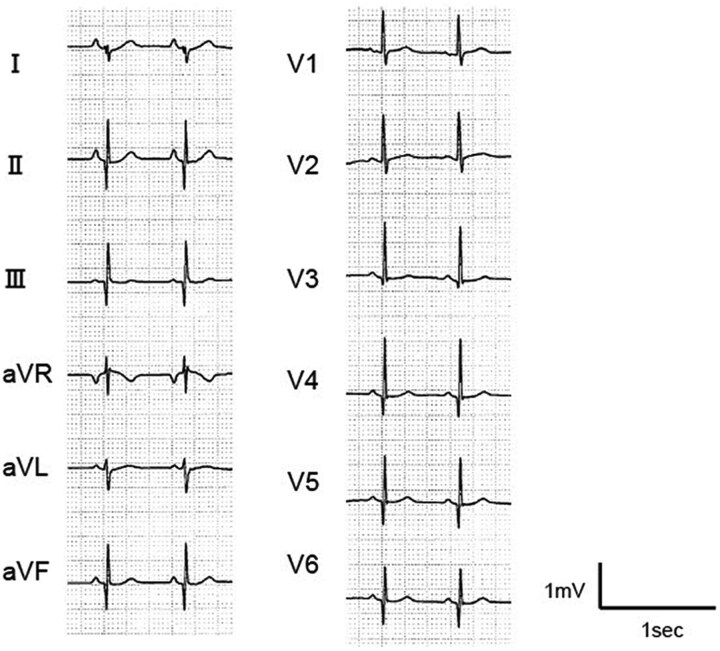
A representative steady-state electrocardiogram. Heart rate of 91 b.p.m. and a corrected QT interval of 362 ms, indicating mild QT shortening.

At the age of 4 years and 2 months, she experienced an initial transient HF, which resolved within a few days. Two months later, she experienced a recurrent transient HF with similar clinical course. Notably, extreme sinus bradycardia can precede and trigger HF. Therefore, she started cilostazol (5 mg/kg/day), and her baseline heart rate (HR) increased from 70 to 90 b.p.m. *Figure 2* is a representative electrocardiogram showing mild QT shortening, with corrected QT (QTc) interval of 362 ms. Despite administering preventive cilostazol therapy, the transient HF recurred two times.

**Figure 3:**
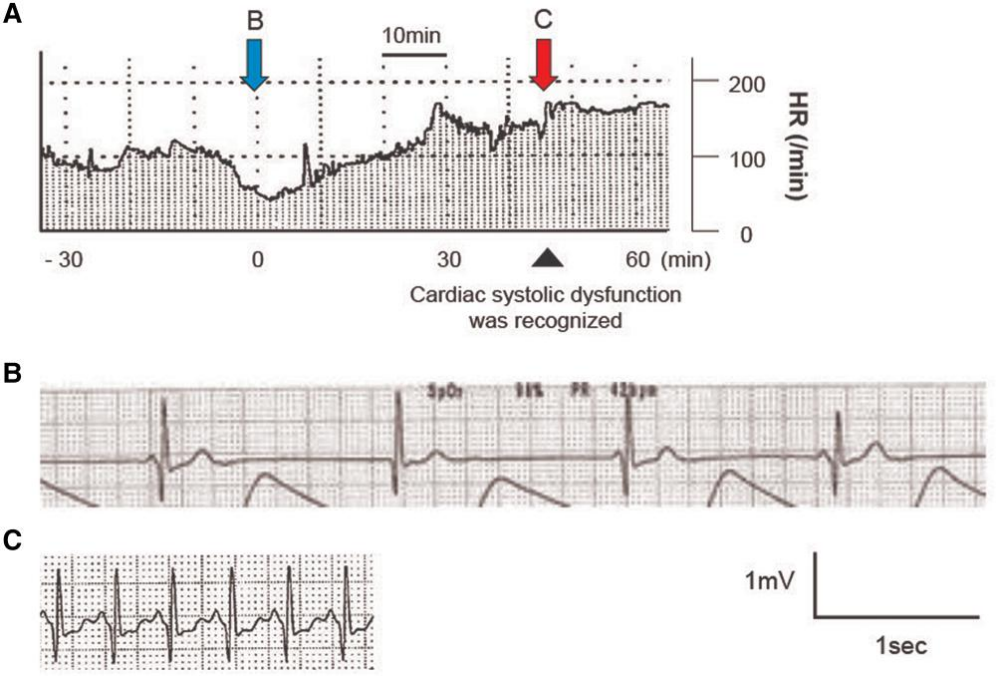
(*A*) Heart rate monitoring around the onset of transient heart failure; (*B*) atrioventricular dissociation due to sinus bradycardia before the onset of transient heart failure; (*C*) sinus tachycardia and ST-segment depression during transient heart failure. No ventricular arrhythmias were observed.

**Figure 4:**
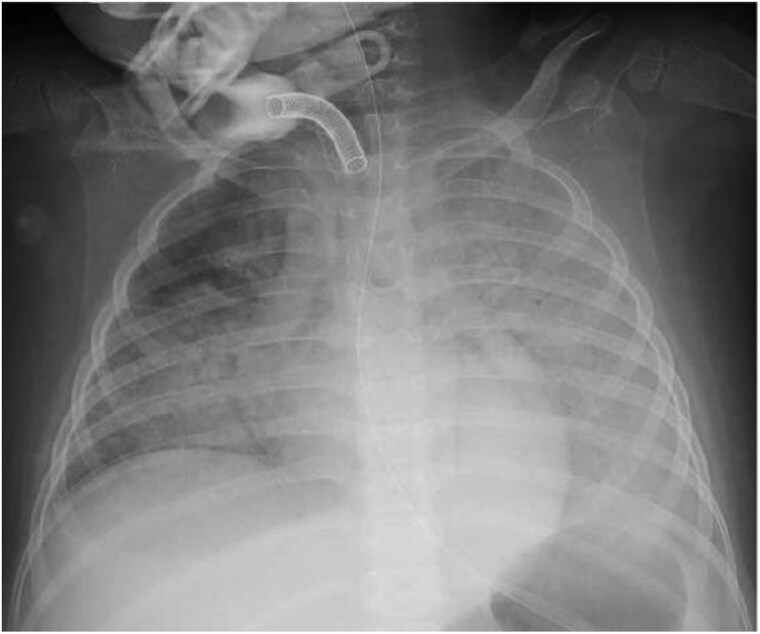
Chest radiograph taken during the onset of transient heart failure. Severe pulmonary congestion suggestive of heart failure was observed.

**Figure 5:**
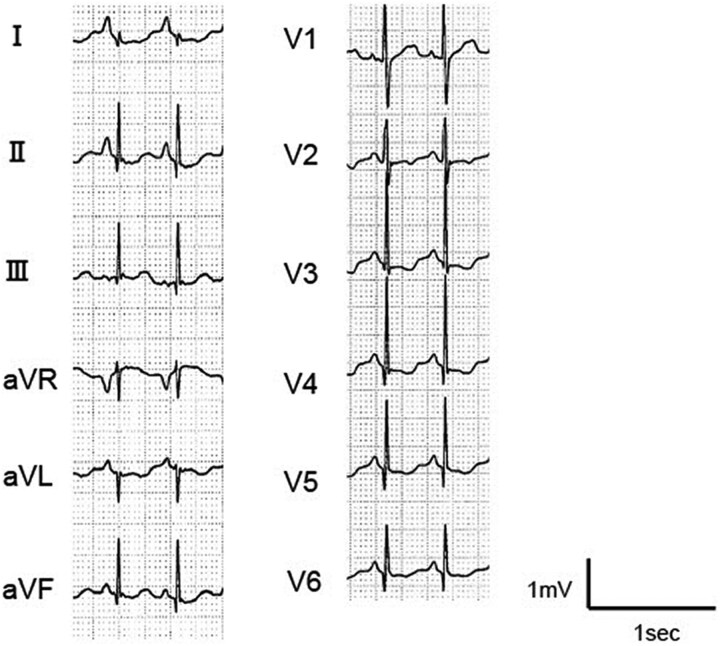
Electrocardiogram during the onset of transient heart failure. It shows extensive ST-segment depression of the sagging type.

The details of the fourth event were as follows: at 4 years and 9 months of age, she was hospitalized because of aspiration pneumonia. During recovery phase, sudden sinus tachycardia accompanied by seizures was followed by atrioventricular dissociation due to severe sinus bradycardia with an HR of <40 b.p.m. (*Figure 3A* and *B*). When her HR gradually rose to 170 b.p.m. 45 min after the onset of bradycardia (*Figure. 3C*), she expectorated frothy blood-tinged sputum. Radiography (*Figure 4*) and echocardiography indicated acute HF. Although she did not have hypotension, cold extremities and metabolic acidosis accompanied by elevated lactate levels were observed. Since these findings suggested low cardiac output, dobutamine (3 μg/kg/min) was initiated. Left ventricular ejection fraction was reduced to 45% (see Supplementary material online, *Video S1*), but improved to 66% the next day (see Supplementary material online, *Video S2*), with a maximum brain natriuretic peptide (BNP) concentration of 485.8 pg/mL. A 12-lead electrocardiogram showed extensive ST-segment depression (the sagging type; *Figure 5*), which gradually normalized across several days. To prevent transient HF, mexiletine was administered at 75 mg/day (5 mg/kg) and then increased to 150 mg/day (10 mg/kg), while cilostazol was discontinued. The preventive mexiletine therapy was approved by the institutional review boards. She is now 6 years and 6 months of age. Following the initiation of the mexiletine therapy, she did not experience any HF event except when she could not take mexiletine because of vomiting. Her HR is approximately 70 b.p.m., and there have been no remarkable changes in ECG findings, including T-wave morphology and QTc interval. No apparent excessive bradycardia has been documented, possibly because her seizures are now well controlled.

## Discussion

Herein, to our knowledge, we indicated for the first time that mexiletine would suppress transient HF in a child having polymicrogyria with a specific *ATP1A3* variant.

Among *ATP1A3*-related disorders, AHC is the most prevalent and has been the most comprehensively investigated. Consistent with our case, AHC patients have been reported to exhibit a short QTc interval.^8^ Some of these patients also predispose to fatal ventricular arrhythmias (VAs) preceded by bradycardia, and cardiomyocytes derived from their induced pluripotent stem cells exhibited delayed afterdepolarizations (DADs).^8^ In our patient, although VAs did not occur, transient HF preceded by bradycardia was observed. Notably, including our case, transient HF has been reported only in variants associated with polymicrogyria, particularly those located within the region interacting with the β-subunit.^4^

In our case, we selected mexiletine for preventing transient HF based on the following presumed pathophysiological mechanism.

Initially, the LOF effect of Na^+^/K^+^ ATPase can reduce Na^+^ extrusion, culminating in intracellular high Na^+^ concentrations. Subsequently, NCX can produce a Ca^2+^ accumulation.^9^ In the steady state, intracellular Ca^2+^ would be increased in *ATP1A3*-related polymicrogyria, similar to that indicated in an AHC mouse model.^3^

Next, consistent with a previous study,^6^ bradycardia preceded transient HF in our case, indicating bradycardia may act as a trigger for transient HF. An augmentation of I_Na, L_ induced by bradycardia^10^ could increase the concentration of intracellular Na^+^ and subsequent Ca^2+^ overload via NCX. Notably, VAs preceded by bradycardia and DADs observed in AHC,^8^ as described above, may reflect this mechanism. In addition, the previous experiment conducted using a mouse model with a heterozygous *ATP1A3* variant concluded that sinus bradycardia and sinus arrest presented while inducing seizures.^2^ Thus, extreme sinus bradycardia complicated with seizures can be a characteristic feature of *ATP1A3*-related disorders.

Finally, the intracellular Ca^2+^ overload would cause myocardial stunning.^11^ Herein, the clinical features, including the sudden onset of myocardial dysfunction with ST-T segment depression and its recovery over several days, were consistent with myocardial stunning. We speculate that the mechanism of this myocardial stunning may overlap with that of stress-induced cardiomyopathy, although the triggers may differ.

Mexiletine is a Na^+^ channel blocker that preferentially blocks I_Na, L_, with no considerable effect on L-type Ca^2+^ channels, but decreases Ca^2+^ accumulation through the NCX mechanism.^12^ Moreover, mexiletine has limited electrophysiological effects on sinus and atrioventricular nodes.^12^ Flunarizine, a T-type Ca^2+^ channel blocker, is effective for AHC.^3^ When targeting cardiomyocytes, verapamil, an L-type Ca^2+^ channel blocker, may prevent transient HF in polymicrogyria; however, we did not use it because it could contribute to bradycardia, thereby triggering transient HF. Therefore, we selected mexiletine as an upstream therapy. Mexiletine was administered safely, successfully preventing transient HF in our case.

*ATP1A3*-related polymicrogyria is a recently proposed disorder potentially with undiagnosed patients. The *ATP1A3* gene should be examined in a patient having polymicrogyria with transient HF, and mexiletine can be one of the key drugs to treat such patients.

## Lead author biography


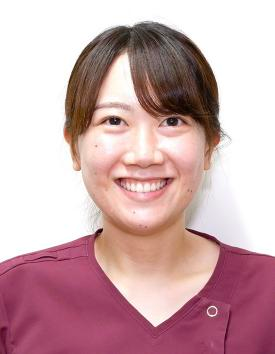


Mai Aida graduated from Niigata University in Japan in 2021. She belongs to the Department of Pediatrics, Niigata University, and she currently works at the Department of Pediatrics, Nagaoka Red Cross Hospital.

## Supplementary Material

ytag106_Supplementary_Data

## Data Availability

The data underlying this article are available in the article and in the online Supplementary material.

## Notes

**Consent:** Written informed consent was obtained from the parents of the patient for publication of this case report in line with COPE guidance.
